# Synthesis and characterization of PLGA/HAP scaffolds with DNA-functionalised calcium phosphate nanoparticles for bone tissue engineering

**DOI:** 10.1007/s10856-020-06442-1

**Published:** 2020-11-02

**Authors:** Viktoriya Sokolova, Kathrin Kostka, K. T. Shalumon, Oleg Prymak, Jyh-Ping Chen, Matthias Epple

**Affiliations:** 1 Inorganic Chemistry and Center for Nanointegration Duisburg-Essen (CeNIDE), University of Duisburg-Essen, Universitaetsstr. 5-7, 45117 Essen, Germany; 2 Department of Chemical and Materials Engineering, Chang Gung University, Kweishan, Taoyuan, 333 Taiwan; 3 Inter University Centre for Nanomaterials and Devices, Cochin University of Science and Technology, Cochin, Kerala 682022 India; 4 Department of Plastic and Reconstructive Surgery and Craniofacial Research Center, Chang Gung Memorial Hospital at Linkou, Collage of Medicine, Chang Gung University, Kwei-San, Taoyuan, 33305 Taiwan

## Abstract

Porous scaffolds of poly(lactide-co-glycolide) (PLGA; 85:15) and nano-hydroxyapatite (nHAP) were prepared by an emulsion-precipitation procedure from uniform PLGA–nHAP spheres (150–250 µm diameter). These spheres were then thermally sintered at 83 °C to porous scaffolds that can serve for bone tissue engineering or for bone substitution. The base materials PLGA and nHAP and the PLGA–nHAP scaffolds were extensively characterized by X-ray powder diffraction, infrared spectroscopy, thermogravimetry, differential scanning calorimetry, and scanning electron microscopy. The scaffold porosity was about 50 vol% as determined by relating mass and volume of the scaffolds, together with the computed density of the solid phase (PLGA–nHAP). The cultivation of HeLa cells demonstrated their high cytocompatibility. In combination with DNA-loaded calcium phosphate nanoparticles, they showed a good activity of gene transfection with enhanced green fluorescent protein (EGFP) as model protein. This is expected enhance bone growth around an implanted scaffold or inside a scaffold for tissue engineering.

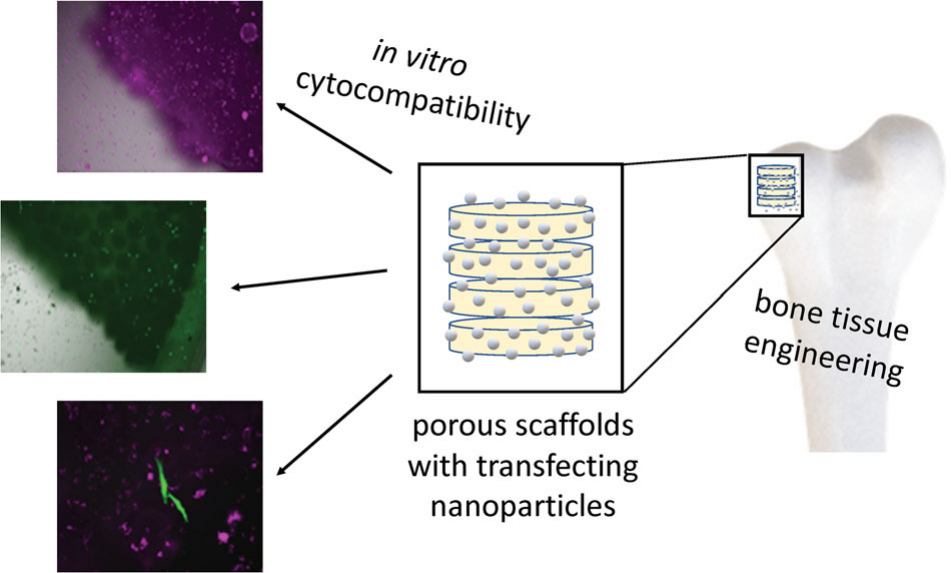

## Introduction

For the regeneration of new bone tissue, the development of a suitable three-dimensional (3D) environment is essential to allow better cell growth and proliferation. Critical properties required for an artificial 3D scaffold, mimicking the extracellular matrix, are non toxicity, biodegradability, porosity, osteoconductivity, and mechanical stability. Various techniques for the fabrication of 3D scaffolds for an enhanced regeneration process have been described [1, 2]. 3D-printing, bioprinting, freeze-drying, or lyophilisation are all important methods to produce porous 3D scaffolds [3–7]. Phase separation and gas foaming are other methods to prepare 3D scaffolds from emulsions and molten polymer solutions, respectively [8, 9]. The self-assembly of smaller objects is another efficient way to develop scaffolds with adequate degradability and regeneration capacity [10–12].

Finally, a versatile option is the fusion of polymer microspheres to 3D scaffolds. For the fabrication of such microsphere-based scaffolds, the most common method is sintering by thermal fusion at elevated temperature. Sintered microsphere scaffolds have been applied for drug delivery and for tissue regeneration therapies, including bone regeneration [13], ligament repair and enhanced vascularization [14], and periodontal regeneration [15]. Apart from scaffold morphology and processing, it is the scaffold composition that eventually determines the regeneration potential. Biodegradable polyesters like polyglycolide and polylactide have a long tradition as scaffolds and surgical material [16–19]. Poly(lactide-*co*-glycolide) (PLGA) is an approved biocompatible and biodegradable polymer. Its softening temperature (glass transition temperature) between 40 and 60 °C is well suited for the fabrication process as it will not damage the incorporated nanoparticles and biomolecules [20–24].

Proteins and growth factors have a high potential to induce tissue regeneration. The delivery of proteins in tissue engineering can be realized with a combination of functionalised nanoparticles, cells, and scaffolds [25–27]. On the other hand, proteins can be synthesized by cells after the successful delivery of plasmids which encode specific proteins, i.e., by transfection [28]. Such a gene delivery is an efficient way to control the cellular response and to generate tissue-specific functional scaffolds. Plasmid DNA delivery was shown to successfully improve the regeneration of bone [29], cartilage [30], skin [31], tendons, muscles [32], and nerves [33]. Polymers, lipids, and inorganic nanoparticles like calcium phosphate, are also capable to act as efficient carriers for gene delivery [34]. Calcium phosphate nanoparticles occur in nature as biominerals and therefore have a high biocompatibility, osteoconductivity, and good biodegradability [35]. They have been applied for different biological and medical applications, e.g., for the transport of biomolecules, gene silencing, transfection, imaging, and immunization [28, 36–46].

Here, we demonstrate how microsphere-based polymer–hydroxyapatite scaffolds are prepared and used for the delivery of DNA-loaded calcium phosphate nanoparticles for in situ cell transfection.

## Materials and methods

### Instruments

Scanning electron microscopy (SEM) was performed with an ESEM Quanta 400 instrument (ThermoFisher) with gold/palladium-sputtered samples. X-ray powder diffraction (XRD) was done with a Bruker D8 Advance diffractometer in Bragg–Brentano geometry (Cu Kα radiation, *λ* = 1.54 Å; 40 kV; 40 mA) and subsequent Rietveld refinement with the Bruker software TOPAS 5.0. The ICSD pattern for hexagonal hydroxyapatite (#09-0432) was used as reference. Dynamic light scattering (DLS) and zeta potential determinations were performed with a Zetasizer Nano ZS instrument (Malvern, laser *λ* = 633 nm) with the Smoluchowski approximation, using the data from the Malvern software. All given particle size data refer to scattering intensity distributions (*z*-average). Ultracentrifugation was performed at 25 °C with a Sorvall WX Ultra Series centrifuge (ThermoElectron Corporation, Schwerte, Germany). UV–Vis absorption spectra were measured with a DS-11 FX+ spectrophotometer (“Nanodrop”, DeNovix, Delaware, USA). The concentration of calcium was determined by atomic absorption spectroscopy (AAS) with an M-Series AA spectrometer (ThermoElectron Corporation). Prior to the AAS measurement, an aliquot of the calcium phosphate nanoparticle dispersion was dissolved with concentrated HNO_3_. Freeze-drying (lyophilization) was done with a Christ Alpha 2–4 LSC instrument.

The transfection efficiency and the uptake studies were performed with transmission light microscopy and fluorescence microscopy. A Keyence Biorevo BZ-9000 instrument (Osaka, Japan), equipped with filters for TRITC (EX 540/25, DM 565, BA 605/55); FITC (EX 465/95, DM 505, BA 515/55), and Cy5 (EX 628/40, DM 660, EM 692/40) with 4×, 10×, and 20× objectives was used. All images were recorded with the BZ-II viewer software and further processed with the BZ-II analyser software.

Thermogravimetric analysis (TGA) was performed with a Netzsch STA-449 F3 Jupiter instrument. A sample mass between 20 and 30 mg was put into an Al_2_O_3_ crucible and then heated from 20 to 1000 °C at a heating rate of 2 K min^−1^ under dynamic oxygen atmosphere (25 mL min^−1^). Infrared spectroscopy was performed with a Platinum ATR, Bruker Alpha instrument (100 scans per measurement). Differential scanning calorimetry (DSC) was performed with a Netzsch DSC 204 Phoenix instrument (sample mass 4–5 mg; Al crucible; heating rate 2 K min^−1^; 30–400 °C).

### Chemicals

The following chemicals were used: Branched poly(ethyleneimine) (PEI; *M* = 25 kDa; Sigma-Aldrich, USA), poly(ethylenimine)-Cy5 (PEI-Cy5; *M* = 25 kDa; Surflay, Germany), calcium lactate (p.a.; Merck), diammonium hydrogenphosphate (p.a.; Merck), tetraethoxysilane (TEOS; 98%, Sigma-Aldrich), and ammonia solution (30–33%, Carl Roth). Calcium hydrogen phosphate (CaHPO_4_·2 H_2_O), sodium hydroxide (NaOH), and poly(vinyl alcohol) (PVA) were purchased from Sigma-Aldrich. Poly(lactide-*co*-glycolide) (PLGA) with a molar ratio of 85:15 lactide to glycolide was obtained from Green Square Materials Inc. (Taiwan). Calcium carbonate was obtained from Scharlau Chemie (Spain). Dichloromethane was obtained from Alfa Aesar (Ward Hill, USA). Ultrapure water (Purelab ultra instrument from ELGA) was used for all preparations unless otherwise noted. All chemicals were of analytical grade and used without further purification.

For transfection experiments, the plasmid pcDNA3-eGFP (*M*_w_ = 4,056,360 g mol^−1^) that encodes for eGFP was prepared from *Escherichia coli* with a Nucleobond endotoxin-free plasmid DNA kit (Macherey-Nagel, Dueren, Germany).

### Fabrication of scaffolds

The scaffold fabrication consisted of three stages, i.e., the nano-hydroxyapatite (nHAP) preparation, the nHAP-PLGA-microsphere preparation, and finally the preparation of the macroscopic scaffold.

Hydroxyapatite nanoparticles were prepared by a precursor precipitation method, where CaHPO_4_·2 H_2_O and CaCO_3_ were reacted [47, 48]. This leads to a partial substitution of phosphate by carbonate due to atmospheric carbon dioxide.

An emulsion-solvent evaporation method was used for the preparation of PLGA/nHAP microspheres [10, 49]. A set of sieves with different pore diameter ranging from 88 to 500 µm was used to separate the microspheres. Microspheres with a diameter between 150 and 250 µm were used for scaffold preparation. The microspheres were stored in a vacuum desiccator until further processing.

PLGA microspheres containing 5, 10, 20, or 40 wt% nHAP, respectively, were then filled into prefabricated disc-shaped stainless-steel molds with 2.4 mm^2^ cavities. After filling the cavities, the molds were tightened with screws and closed with rectangular closing lids, without disturbing the microsphere packing. The microspheres were surface sintered (cross-linked) at 83 °C for 90 min and then allowed to freely cool to ambient temperature. The scaffolds were stored in a desiccator until further characterization.

### Synthesis of functionalized calcium phosphate nanoparticles

The synthesis of plasmid DNA-loaded calcium phosphate nanoparticles was carried out as reported earlier [28]. Briefly, calcium phosphate nanoparticles were stabilized with the cationic polyelectrolyte PEI or PEI-Cy5 and then loaded with plasmid DNA. The nanoparticles were then coated with a silica shell to protect the plasmid from nucleases [50]. For storage, the dispersions were freeze dried after isolation by ultracentrifugation [28]. The particles were easily redispersible in pure water immediately before further use.

The concentration of Ca^2+^ in the nanoparticle dispersion was determined by AAS and converted to calcium phosphate, assuming the stoichiometry of hydroxyapatite, Ca_5_(PO_4_)_3_OH. The nanoparticle concentration in 1 mL of the colloidal dispersion was computed from the calcium phosphate concentration with the density of hydroxyapatite (3140 kg m^−3^), assuming spherical particles (diameter taken from the SEM images; see ref. [51]. for a typical calculation). The concentration of unbound pDNA-eGFP was measured with a DS-11 FX+ spectrophotometer (DeNovix, Delaware, USA) in the supernatant after the purification.

### Cell culture

HeLa cells (human cervix carcinoma cell line) were cultivated in Dulbecco’s modified Eagle’s medium (DMEM) supplemented with 10% fetal calf serum (FCS; Thermo Fischer), and antibiotics at 37 °C under 5% CO_2_ atmosphere. One day before the incubation with nanoparticles, the cells were trypsinized and seeded in 24-well plates.

### Cell uptake and transfection studies

For cell uptake and transfection studies, HeLa cells were trypsinized and seeded in a 48-well plate with a density of 12.5 × 10^3^ cells per well in 250 µL medium (DMEM with FCS) in 48-well plates. After 24 h, 10 μL of the nanoparticle dispersions were added to cell cultures into each well. After 48 h, the whole cell culture medium was removed with a pipette and the cells were washed three times with PBS. The cells were then studied by fluorescence microscopy. The transfection efficiency was determined by fluorescence microscopy and defined as the ratio of the number of cells in which enhanced green fluorescent protein (EGFP) was expressed to the total number of cells [52].

### Cell uptake and transfection studies in scaffolds

For cell uptake and transfection studies in scaffolds, the following steps were performed. HeLa cells were trypsinized and seeded at a density of 12.5 × 10^3^ cells per well onto different scaffolds in a 48-well plate in 250 µL medium in the 48-well plates in DMEM with FCS. After 2 h of incubation (cells adsorbed on the surface of scaffolds), 10 μL of the nanoparticle dispersion was added per well. After 48 h, the whole cell culture medium was removed and the cells with scaffolds were washed three times with PBS. The cells were then studied by fluorescence microscopy.

## Results and discussion

**Fig. 1:**
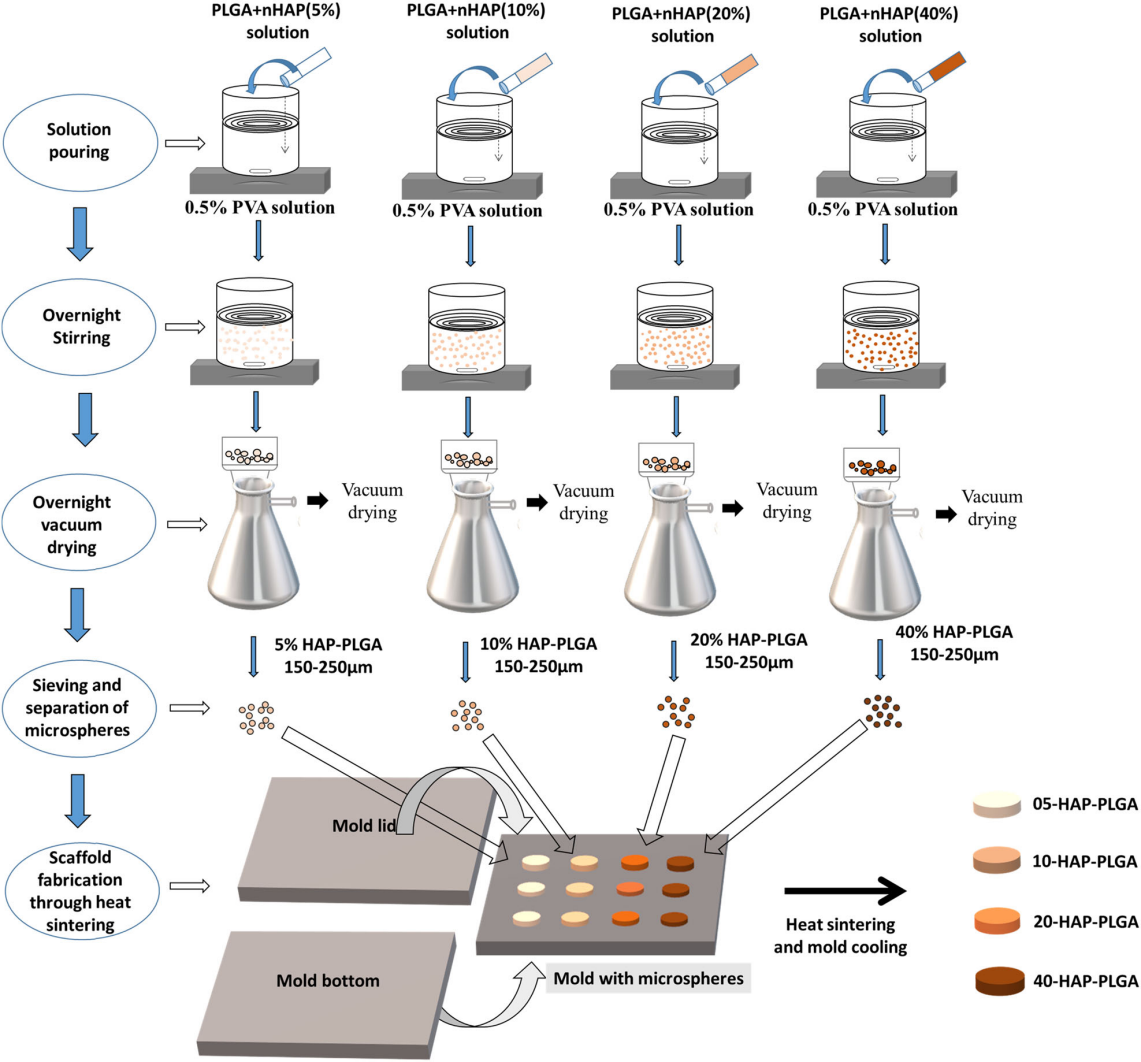
Schematic representation of the preparation of PLGA–nHAP microspheres with 5, 10, 20, and 40 wt% nHAP content and their respective macroporous scaffolds by various steps, i.e., solvent pouring, overnight stirring, drying, sieving, and thermal sintering

PLGA–nHAP scaffolds were prepared by an emulsion-precipitation procedure. The microspheres were then thermally surface sintered at 83 °C to porous macroscopic scaffolds. The full synthetic procedure and the scaffold fabrication are depicted in Fig. 1.

**Fig. 2:**
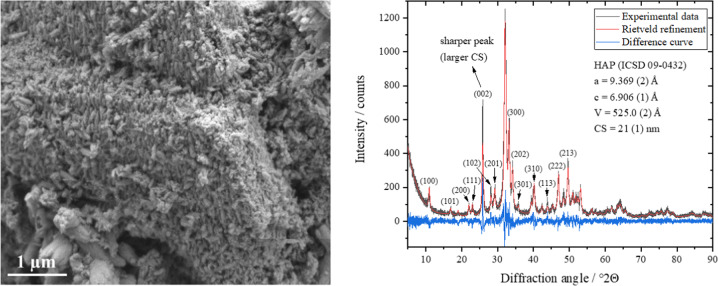
Representative scanning electron microscopy image (left) and Rietveld refinement (right) of the HAP nanocrystals (nHAP) with the calculated lattice parameters *a* and *c*, and the average crystallite size (CS) after isotropic refinement. The diffraction peaks in the [001] *c*-direction were sharper, indicating larger crystallites. Standard deviations are given in parentheses

First, the nano-hydroxyapatite was characterized. The initial HAP powder with a particle size of about 5–10 µm consisted of elongated nanocrystalline HAP crystals with particle and crystallite sizes of about 20–30 nm as determined by SEM and XRD, respectively (Fig. 2). The narrow (002) diffraction peak indicated a larger crystallite size along the *c*-axis and confirmed the elongated nature of HAP nanoparticles in the crystallographic *c*-direction. To quantify the anisotropic dependency of the crystallite sizes on different crystallographic directions in the HAP nanocrystals, an individual peak profile refinement was performed, and the crystallite size as function of the crystallographic direction ([hkl] Miller index) was determined (Table 1). The average crystallite size was 21 nm, except for the [00l] *c*-direction, as indicated by the Miller indices (002), (102), and (202) with a crystallite size of 37, 27, and 25 nm, respectively. This confirmed the rod-like nature of the HAP nanocrystals in agreement with the SEM results (Fig. 2).

**Table 1:** Anisotropic crystallite size (CS) in different crystallographic directions in nHAP with (*hkl*) the corresponding Miller indices, calculated from X-ray powder diffraction (Rietveld refinement)

(hkl)	(100)	(101)	(200)	(111)	(002)	(102)	(201)	(300)	(202)	(301)	(310)	(113)	(222)	(213)
2*Θ*°	10.93	16.89	21.95	22.98	25.85	28.14	29.17	33.19	34.16	35.72	40.15	43.90	46.96	49.61
CS/nm	22	21	21	20	37	27	18	19	25	13	15	27	22	22

**Fig. 3:**
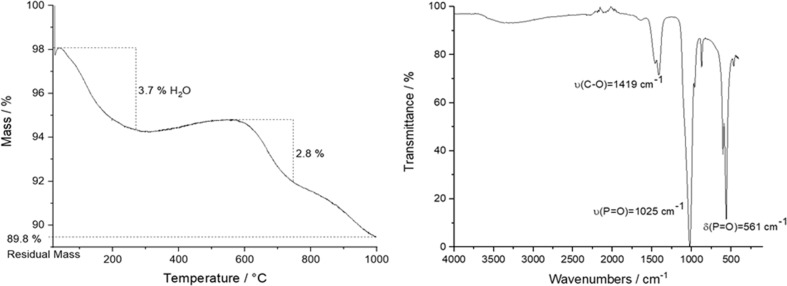
Thermogravimetry (left) and infrared spectroscopy (IR) (right) of the initial HAP powder

The presence of atmospheric carbon dioxide during the precipitation led to a partial substitution of phosphate by carbonate groups (B-type carbonated apatite), making the material more similar to bone mineral [53–55]. TGA and IR spectroscopy were applied for the quantification of carbonate (Fig. 3). TGA showed two main regions during the sample heating to 1000 °C. The first (25–200 °C) with a mass loss of 3.7% is related to the release of adsorbed or incorporated water, the second (500–1000 °C) with about 2.8% is due to the release of CO_2_ from carbonated apatite [56]. This corresponds to about 3.8 wt% incorporated carbonate. IR spectroscopy showed typical broad bands for the phosphate groups at 1025 and 561 cm^−1^ and a split C–O band at 1460/1419 cm^–1^ that is responsible for B-type carbonate substitution [55].

**Fig. 4:**
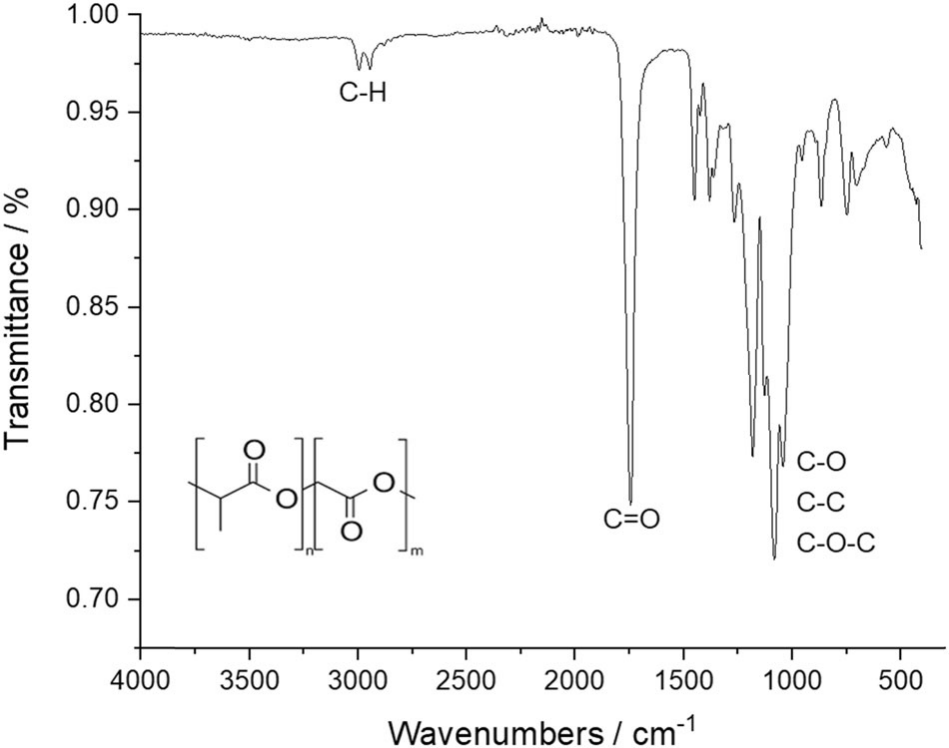
Representative IR spectrum of the initial PLGA (85:15) powder

The PLGA base material showed the expected bands of the functional C–O and C–H groups in IR spectroscopy and no impurities (Fig. 4) [57].

**Fig. 5:**
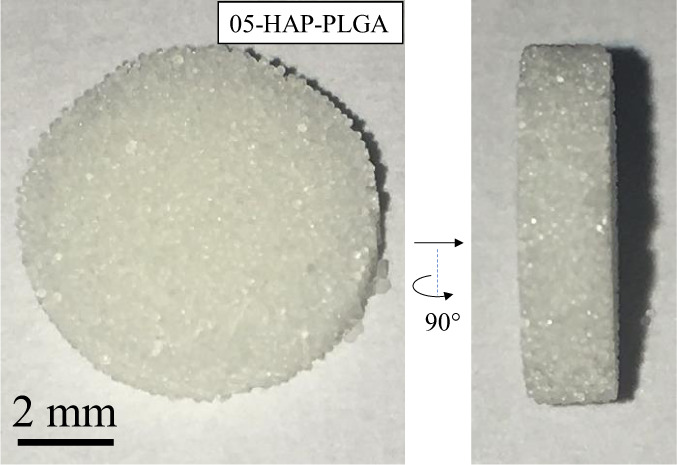
Image of a 05-HAP–PLGA scaffold with about 48% porosity, seen from the top and from the side. The surface-sintered PLGA–HAP spheres form a highly porous structure

The PLGA–nHAP microparticles were then thermally surface sintered to porous scaffolds. Figure 5 shows an image of such a sintered porous PLGA/HAP scaffold.

**Fig. 6:**
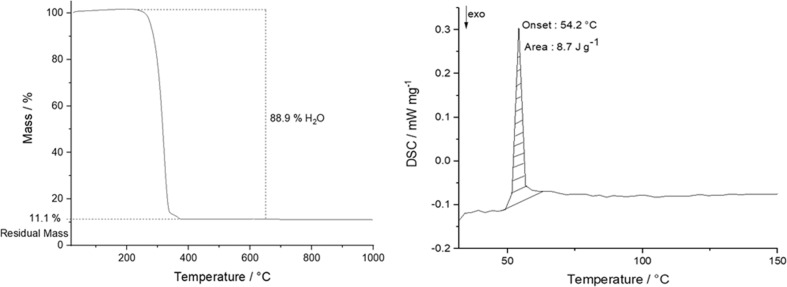
Representative thermogravimetry (left) and DSC curve (right) analysis of a 10-HAP–PLGA scaffold

The PLGA/HAP scaffolds with 5, 10, 20, and 40 wt% hydroxyapatite, respectively, were thoroughly characterized as well. The amount of hydroxyapatite (HAP) in the scaffolds was determined by thermogravimetry (TGA; Fig. 6), giving 7.6, 11.1, 14.9, and 27.8 wt% HAP, respectively, i.e., close to the nominal composition. In the following, the samples are denoted with their nominal composition, e.g., as 10-HAP–PLGA for 10 wt% HAP. DSC analyses of the scaffolds (Fig. 6) showed an endothermic peak at 54 °C, confirming the typical glass transition temperature of PLGA in the range of 40–60 °C [57].

**Fig. 7:**
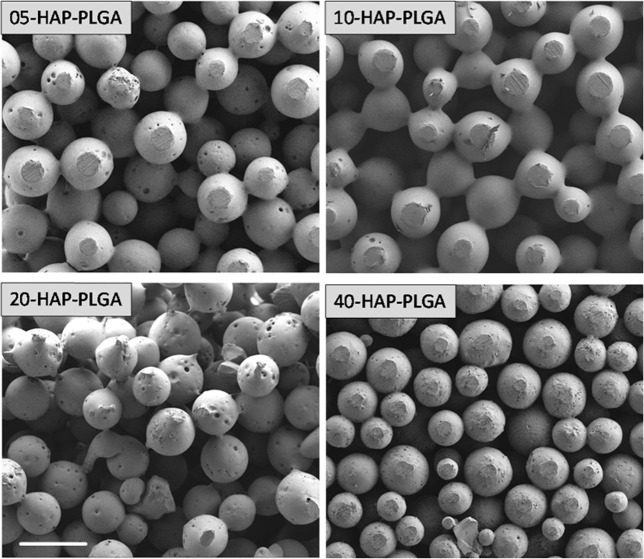
Representative scanning electron microscopy images of the surface-sintered HAP/PLGA scaffolds (5, 10, 20, and 40 wt% HAP). The spheres and scaffolds are uniform, independent from the HAP content. Scale bar 300 µm

**Fig. 8:**
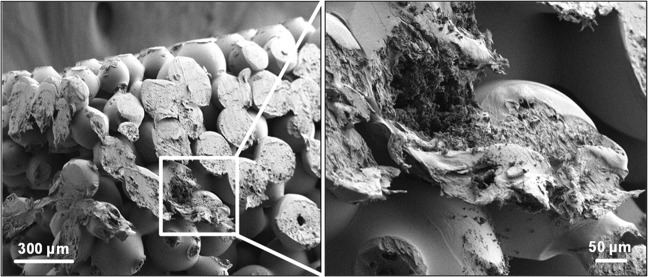
Representative scanning electron microscopy images of a fracture surface of a 10-HAP–PLGA scaffold at low (left) and high magnification (right). The scaffolds with different nHAP loading gave very similar images (data not shown)

SEM showed that the surface-sintered scaffolds contained PLGA spheres with interconnected porosity and a diameter of about 200 µm (Fig. 7). The spheres were comparable in size for all scaffolds and showed the successful incorporation of nanocrystalline HAP crystals and the interconnection of the spheres (Fig. 8). Energy-dispersive X-ray spectroscopy (EDX) confirmed that the small crystals consisted of calcium phosphate (Ca and P), but due to the uneven sample surface it was not possible to determine the molar Ca:P ratio. Other EDX signals were due to carbon and oxygen from PLGA and nHAP.

By measuring size and weight of the disc-shaped scaffolds together with their actual composition as derived by thermogravimetry, we computed the porosity to 48, 49, 46, and 61 vol% for 5, 10, 20, and 40 wt% HAP. We used the X-ray density of nHAP (3.17 g cm^−3^) and the density of pure PLGA (1.34 g cm^–3^) as basis to compute the absolute scaffold density. Together with the effective density of the porous scaffold, this gave the scaffold porosity. The mechanical properties of such porous scaffolds were in the range of 50–70 MPa (Young’s modulus) [10, 47].

**Fig. 9:**
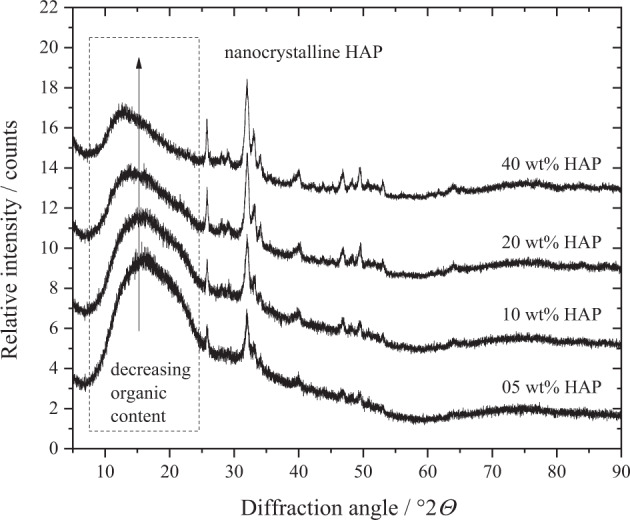
X-ray powder diffractograms of HAP–PLGA scaffolds (5, 10, 20, and 40 wt% HAP) with an amorphous halo around 15 °2*Θ* for PLGA and broad diffraction peaks for nanocrystalline nHAP

The XRD analysis of the PLGA/HAP scaffolds showed broad reflection peaks for nanocrystalline HAP for all samples, demonstrating the successful and unchanged incorporation of nHAP (Fig. 9). The lattice parameters (*a* = 9.39 Å, *c* = 6.91 Å) and crystallite sizes (18 ± 1 nm) agreed well with the initial nHAP powder. The amorphous halo caused by PLGA in the region of 10–25 °2*Θ* decreased with increasing inorganic content of nHAP from 5 to 40 wt% as expected. The incorporated HAP showed a contraction in *a* direction and an extension in *c* direction compared to pure HAP (ICSD database; *a* = 9.42 and *c* = 6.88 Å). This can be explained by the incorporation carbonate on the phosphate position, leading to B-type carbonated apatite [54, 58]. The fact that the crystallographic parameters of nHAP did not change significantly after incorporation and sintering confirms the successful incorporation in the original form into the scaffold.

**Fig. 10:**
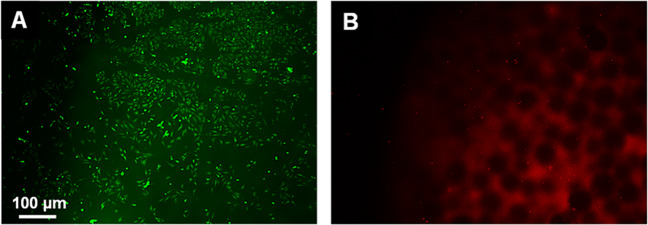
Representative fluorescence microscopy images of a 10-HAP–PLGA scaffold, seeded with HeLa cells (after live/dead assay). Living cells are stained green **a**, dead cells are stained red **b**. Note the red autofluorescence of the scaffold

The cytocompatibility of the sintered scaffolds was shown by seeding HeLa cells on their surface. In Fig. 10, fluorescence microscopy images of an nHAP/PLGA scaffold (10 wt% nHAP) seeded with HeLa cells, followed by a live/dead assay are shown. Most cells were living (green fluorescentwere present, demonstrating the high biocompatibility. In the red channel, we observed a small number of red cells (indicating dead cells), but also a considerable autofluorescence of the scaffold.

**Fig. 11:**
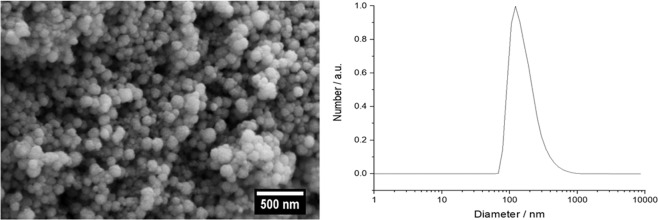
Representative scanning electron microscopy (left) and dynamic light scattering analysis of CaP/PEI/pEGFP/SiO_2_ nanoparticles (right). CaP/PEI/pEGFP/SiO_2_ and CaP/PEI-Cy5/SiO_2_ nanoparticles gave very similar results (data not shown)

DNA-loaded calcium phosphate nanoparticles were prepared for a transfection from the scaffolds. The particles were fluorescently labeled by PEI-Cy5 and also loaded with the pEGFP plasmid DNA. All calcium phosphate nanoparticles were synthesized and colloidally stabilized by the cationic polyelectrolyte PEI. Three different types of calcium phosphate nanoparticles were prepared and characterized. The first type of fluorescing calcium phosphate nanoparticles was prepared with Cy5-labeled PEI as a fluorescent label to study the cellular uptake by fluorescence microscopy. The second type of nanoparticles (with unlabeled PEI) was loaded with plasmid EGFP-DNA for transfection experiments. The third type of nanoparticles was prepared to simultaneously follow uptake and transfection, i.e., fluorescing nanoparticles (with PEI-Cy5) were loaded with plasmid EGFP-DNA. All nanoparticles were coated with a silica layer to protect the DNA from degradation by nucleases. A spherical morphology of all nanoparticles was observed in SEM, and a good dispersibility was shown by DLS (Fig. 11). All nanoparticle characterization data are given in Table 2.

**Table 2:** Characterization data of the functionalized calcium phosphate nanoparticles

Sample	CaP/PEI-Cy5/SiO_2_	CaP/PEI/pEGFP/SiO_2_	CaP/PEI-Cy5/pEGFP/SiO_2_
Solid core particle diameter by SEM/nm	83	119	94
*V*(one nanoparticle; only CaP)/m^3^	2.99 × 10^–22^	8.82 × 10^–22^	4.35 × 10^−22^
*m*(one nanoparticle; only CaP)/kg	9.40 × 10^–19^	2.77 × 10^–18^	1.36 × 10^−18^
*w*(Ca^2+^) in the dispersion by AAS/kg m^−3^	0.094	0.097	0.089
*w*(Ca_5_(PO_4_)_3_OH) in the dispersion/kg m^−3^	0.236	0.243	0.223
*N*(nanoparticles) in the dispersion/m^−3^	2.51 × 10^17^	8.78 × 10^16^	1.63 × 10^17^
*w*(plasmid) in the dispersion/kg m^–3^	–	0.120	0.122
*N*(plasmid)/m^–3^	–	1.78 × 10^19^	1.81 × 10^19^
*m*(plasmid) per nanoparticle in the dispersion/kg	–	1.37 × 10^−18^	7.46 × 10^−19^
*N*(plasmid) molecules per nanoparticle	–	2.03 × 10^3^	1.11 × 10^3^
weight ratio of plasmid to calcium phosphate	–	1: 0.49	1: 0.55
*w*(PEI-Cy5) in the dispersion/kg m^–3^	0.011	–	0.013
*N*(PEI-Cy5) in the dispersion/m^−3^	2.63 × 10^20^	–	3.04 × 10^20^
*m*(PEI-Cy5) per nanoparticle/kg	4.35 × 10^−^^20^	–	7.71 × 10^−^^20^
*N*(PEI-Cy5) molecules per nanoparticle	1.05 × 10^3^	–	1.86 × 10^3^
Hydrodynamic particle diameter by DLS/nm (*number*)	122	124	212
PDI by DLS	0.31	0.28	0.32
Zeta potential by DLS/mV	+21	+26	+42

The given concentrations refer to the nanoparticle stock solutions which were then diluted and applied in the biological experiments. See experimental part for details on the characterization and the calculation

*PDI* polydispersity index from DLS

**Fig. 12:**
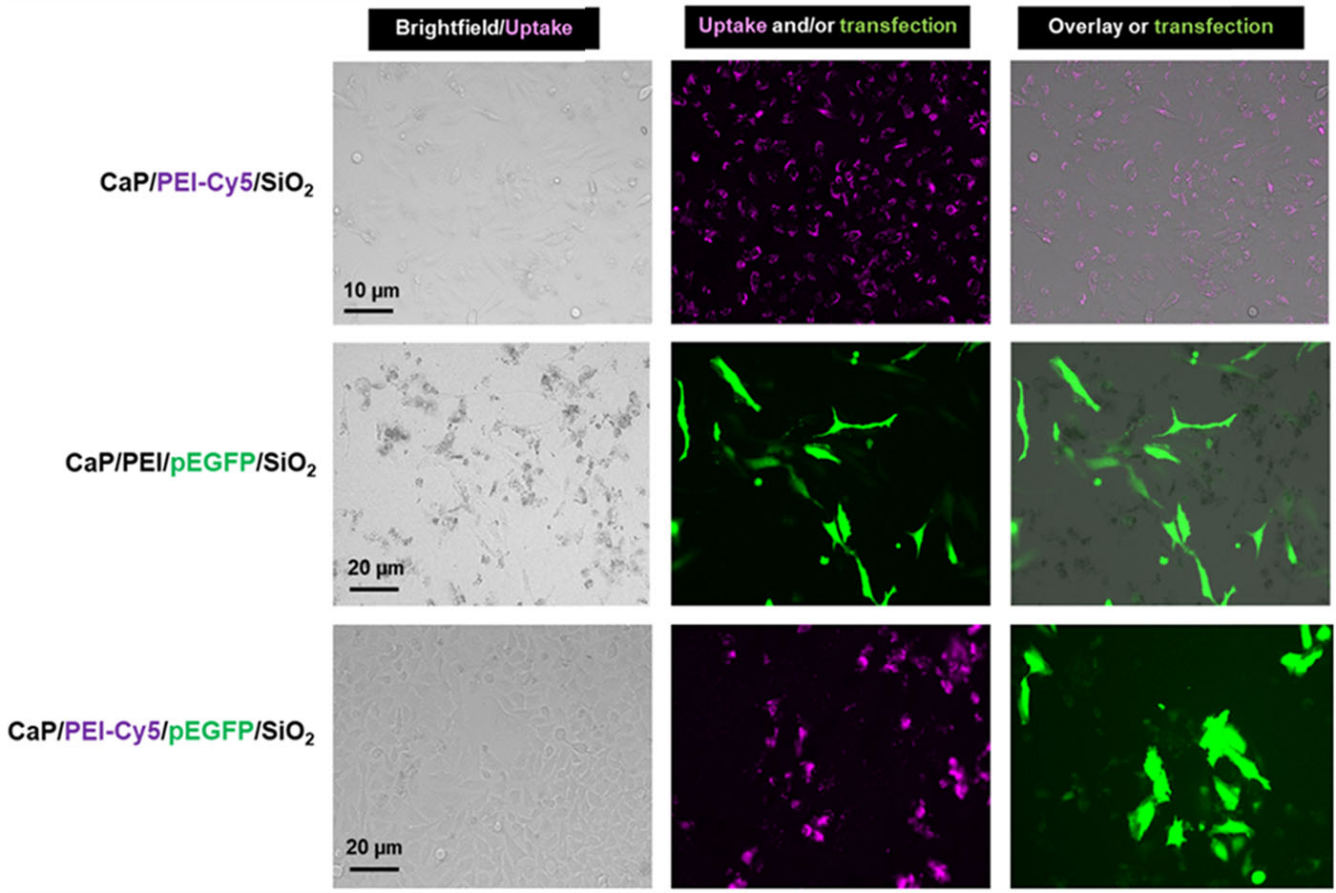
Uptake of CaP/PEI-Cy5/SiO_2_ nanoparticles (top row), transfection with CaP/PEI/pEGFP/SiO_2_ nanoparticles (center row), and uptake and transfection with CaP/PEI-Cy5/pEGFP/SiO_2_ nanoparticles (bottom row) after incubation of HeLa cells for 48 h

The fluorescently labeled nanoparticles were efficiently taken up by HeLa cells. In previous studies we have shown that such calcium phosphate nanoparticles have a low cytotoxicity [28]. The transfection efficiency of DNA-carrying calcium phosphate nanoparticles in HeLa cells after 48 h was about 36% for CaP/PEI/pEGFP/SiO_2_ and 44% for CaP/PEI-Cy5/pEGFP/SiO_2_ nanoparticles (Fig. 12).

**Fig. 13:**
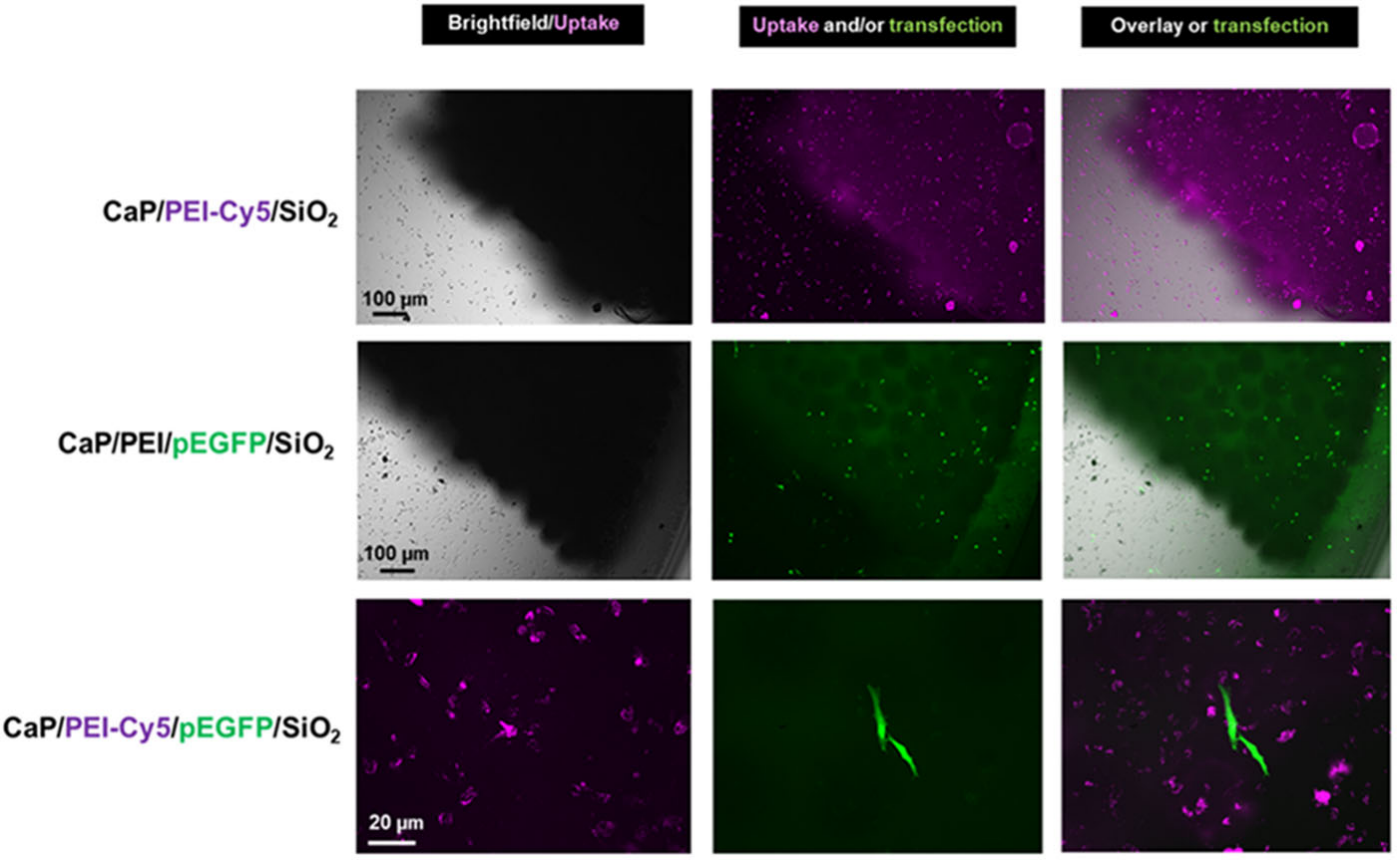
Representative fluorescence microscopy images for the uptake of CaP/PEI-Cy5/SiO_2_ nanoparticles (top row), transfection with CaP/PEI/pEGFP/SiO_2_ nanoparticles (center row) and uptake and transfection with CaP/PEI-Cy5/pEGFP/SiO_2_ nanoparticles (bottom row) after the incubation of scaffolds with HeLa cells for 48 h with nanoparticles on a 40-HAP/PLGA scaffold

In the next step, we studied the uptake and transfection efficiency of the PLGA scaffolds incubated with nanoparticles. HeLa cells were seeded on the top of each scaffold, incubated for 2 h, and the three kinds of nanoparticles were added, respectively. The cells on the scaffolds were cultivated for 48 h. The nanoparticle uptake was studied with fluorescing CaP/PEI-Cy5/SiO_2_ nanoparticles. The nanoparticles were taken up by all cells and were observed in fluorescence microscopy as bright dots distributed in the cells. Non-fluorescing CaP/PEI/pEGFP/SiO_2_ nanoparticles were applied for transfection experiments, where the transfection efficiency was determined by fluorescence microscopy after 48 h of incubation (Fig. 13). In the presence of scaffolds the nanoparticles efficiently delivered pDNA-EGFP into the cells, where pDNA-EGFP induced the production of EGFP. By applying fluorescing CaP/PEI-Cy5/pEGFP/SiO_2_ nanoparticles, loaded with plasmid pDNA-EGFP, we showed that not each cell that took up the nanoparticles was actually transfected. This means that although the nanoparticles crossed the cell membrane and delivered the DNA into the cell, there are other processes (like endosomal escape and DNA degradation by nucleases) which prevent a successful transfection of cells [59–62]. We finally note that all scaffolds were stable and lost neither integrity nor shape during the immersion in cell culture medium.

## Conclusions

Surface-sintered scaffolds consisting of microspheres of poly(lactide-*co*-glycolide) and nano-hydroxyapatite (PLGA–nHAP) can be prepared with high porosity (about 50%) and different loadings of nHAP. The inorganic phase was not changed by incorporation and scaffold sintering as shown by XRD. They are highly cytocompatible and can serve as bioactive scaffolds for a release of DNA-loaded calcium phosphate nanoparticles for local gene transfection. The nanoparticles were taken up by the seeded cells and delivered the DNA plasmid into the cells, leading to an expression of EGFP. For an application as scaffold for bone tissue engineering or as bone substitution material, the combination of the scaffolds with bioactive calcium phosphate nanoparticles with plasmids that encode proteins that stimulate bone growth (like BMP or VEGF) is a promising pathway. Future studies should include the growth of bone cells onto and into the scaffolds, including the measurement of bone growth marker proteins.
